# Protease Inhibitors in Tick Saliva: The Role of Serpins and Cystatins in Tick-host-Pathogen Interaction

**DOI:** 10.3389/fcimb.2017.00216

**Published:** 2017-05-29

**Authors:** Jindřich Chmelař, Jan Kotál, Helena Langhansová, Michail Kotsyfakis

**Affiliations:** ^1^Faculty of Science, University of South Bohemia in České BudějoviceČeské Budějovice, Czechia; ^2^Institute of Parasitology, Biology Center, Czech Academy of SciencesČeské Budějovice, Czechia

**Keywords:** tick-host interaction, immunomodulation, protease inhibitors, serpins, cystatins

## Abstract

The publication of the first tick sialome (salivary gland transcriptome) heralded a new era of research of tick protease inhibitors, which represent important constituents of the proteins secreted via tick saliva into the host. Three major groups of protease inhibitors are secreted into saliva: Kunitz inhibitors, serpins, and cystatins. Kunitz inhibitors are anti-hemostatic agents and tens of proteins with one or more Kunitz domains are known to block host coagulation and/or platelet aggregation. Serpins and cystatins are also anti-hemostatic effectors, but intriguingly, from the translational perspective, also act as pluripotent modulators of the host immune system. Here we focus especially on this latter aspect of protease inhibition by ticks and describe the current knowledge and data on secreted salivary serpins and cystatins and their role in tick-host-pathogen interaction triad. We also discuss the potential therapeutic use of tick protease inhibitors.

## Serpins and cystatins as homeostatic regulators

Proteases (also proteinases or peptidases) are ubiquitous enzymes that cleave proteins to smaller peptides and amino acids. Proteases participate in a range of physiological processes including extracellular digestion, protein degradation, and tissue development (Rawlings and Salvesen, 2013). Relevant to this review, however, is the fact that many proteases, in particular highly substrate-specific endopeptidases, mediate defense and homeostatic processes in both vertebrates and invertebrates. Proteolytic pathways rely on the precise and tightly regulated activation and inhibition of these endopeptidases. As a result of this evolutionary need, many crucial pathophysiological processes are regulated via proteolytic cascades, with notable examples being coagulation of plasma (or haemolymph in arthropods), bacterial wall perforation with complement, or melanization in arthropods (Amara et al., 2008; Tang, 2009; Gulley et al., 2013). Each step involves proteolytic activation of another downstream protease, and all proteases in such cascades usually have their own endogenous inhibitors that balance the system. The role of arthropod protease inhibitors in the defense is supported by the fact that the expression of serpins and cystatins in *Ixodes scapularis* nymphs was attenuated upon infection with *Anaplasma phagocytophilum*, as seen in the transcriptomic data (Ayllon et al., 2015). On the other hand, the expression of protease inhibitors in salivary glands and midguts of adult females differed among individual inhibitors, i.e., some cystatins and serpins were upregulated upon the infection and vice versa (Ayllon et al., 2015). Similar data were collected from *Ixodes ricinus* infected with *Bartonella henselae* (Liu et al., 2014). Therefore, precise involvement of every individual inhibitor in tick infection would have to be evaluated experimentally.

Other intracellular and extracellular processes, such as cytokine activation, phagocytosis, intracellular signaling, and antigen processing, are also dependent on proteolysis (Muller et al., 2012). Serpins and cystatins are the two main superfamilies of endogenous serine and cysteine protease inhibitors involved in the regulation of these processes. It is therefore unsurprising that both groups of inhibitors are well represented in parasites and are important in their interactions with hosts (Schwarz et al., 2012; Meekins et al., 2017). In order to obtain a blood meal, ticks secrete hundreds of different pharmacoactive molecules into the host via their saliva. These molecules have anti-hemostatic, anti-inflammatory, anti-complement and immunomodulatory properties and their function is to overcome or evade host defense mechanisms including immune response (Brossard and Wikel, 2004; Chmelar et al., 2012). Moreover, tick saliva and also several salivary compounds were found to facilitate and enhance the establishment of tick-borne pathogens in the host (Anguita et al., 2002; Pal et al., 2004; Kazimirova and Stibraniova, 2013; Wikel, 2013). Inhibitors of proteases represent the most prominent protein families in tick salivary secretion that are responsible for alteration of many different host defense pathways.

## Serine protease inhibitors in ticks

Four groups of serine protease inhibitors have been described in ticks: Kunitz domain inhibitors, Kazal domain inhibitors, trypsin inhibitor-like cysteine rich domain (TIL) inhibitors, and serpins. Inhibitors with 1–7 Kunitz domains mostly act as anti-hemostatic proteins and form a large multigenic family of secreted salivary proteins in ticks that have probably played a crucial role in the development of tick hematophagy (Corral-Rodriguez et al., 2009; Dai et al., 2012; Schwarz et al., 2014). Moreover, single Kunitz-domain inhibitors in other organisms are involved in ion channel blockade and may play a similar role in ticks (Frazao et al., 2012; Valdes and Moal, 2014). Kazal domain inhibitors are described in hematophagous insects such as mosquitoes and triatomine bugs (Rimphanitchayakit and Tassanakajon, 2010), but they are only rarely reported in ticks, in which their function is still unknown (Zhou et al., 2006a; Mulenga et al., 2007a, 2008). TIL-domain inhibitors represent an interesting group of small inhibitors with a conserved 5-disulphide bridge structure that were first reported in *Apis melifera* (Bania et al., 1999) and have also been detected in ticks (Fogaca et al., 2006; Sasaki et al., 2008). The sequences of over 80 TIL-domain inhibitors have been found in arthropod genomes (Zeng et al., 2014), and the unique features of TIL-domain proteins make them an excellent model for designing novel serine protease inhibitors and antimicrobial peptides (Li et al., 2007).

### Serpins

Serpins form the largest superfamily of protease inhibitors, and they are ubiquitously distributed in nature including viruses and prokaryotes. With over 1,500 members, serpins are the most studied protease inhibitors (Law et al., 2006), also helped by their unique and highly intriguing mechanism of inhibition (Whisstock et al., 2010) and the evolutionary changes that turned inhibitory serpins into non-inhibitory proteins with completely different functions (Law et al., 2006; Silverman et al., 2010). For example, there are 29 inhibitory and seven non-inhibitory serpins in humans and 60 functional serpin genes in mice (Heit et al., 2013). Angiotensinogen is a non-inhibitory serpin that is proteolytically activated by renin into several oligopeptides (angiotensins) that regulate vasoconstriction and blood pressure (Lu et al., 2016). Cortisol and thyroxine-binding proteins (human *SERPINA6* and *SERPINA7*) are also notable serpins that act as major transport proteins for glucocorticoids and progesterone (Carrell and Read, 2016). Inhibitory serpins have very diverse functions depending on their specificity, but their importance is highlighted by the serpinopathies—diseases caused by serpin dysfunction or deficiency (Belorgey et al., 2007). Emphysema, cirrhosis, angioedema, hypertension, and even familial dementia are caused at least in part by serpin dysfunction (Kim et al., 1995; Davis et al., 1999; Ekeowa et al., 2009; Huntington and Li, 2009; Lomas et al., 2016).

Arthropod serpins have mostly immunological and hemostatic functions. Serpins have been shown to regulate haemolymph coagulation, are involved in phenoloxidase system activation in insects, and regulate an immune toll pathway in haemolymph (Kanost, 1999; Gulley et al., 2013; Meekins et al., 2017). Furthermore, in bloodfeeding arthropods, serpins can act as modulators of host hemostasis and/or immune responses. Indeed, several insect serpins act as anti-coagulants, anti-complement proteins and immunosuppressors (Stark and James, 1995, 1998; Colinet et al., 2009; Calvo et al., 2011; Ooi et al., 2015). Serpins are abundant in ticks, and one of their functions is to modulate host immune system. Recent advances in this area have been facilitated by the publication of *I. scapularis* genome (Gulia-Nuss et al., 2016) and several next-generation sequencing transcriptome studies that added tens of unique sequences from different tick species to already existing and long list of tick serpins. In 2009, Mulenga and colleagues found 45 serpins in the genome of *I. scapularis* (Mulenga et al., 2009). Two years earlier, the same group described 17 serpins (Lospins) in *Amblyomma americanum* (Mulenga et al., 2007b). This number was, however, substantially broadened by the combination of several approaches up to approximately 120 serpins (Karim and Ribeiro, 2015; Porter et al., 2015, 2017). In the work of Porter and colleagues (Porter et al., 2015), the authors compare homologous serpins across tick species, showing both conserved and species-specific inhibitors. The conservation seems to be higher in serpins with basic or polar uncharged amino acid residues at P1 site (Porter et al., 2015). Other 32 serpin transcripts from the *Amblyomma* genus were found in *Amblyomma maculatum* (Karim et al., 2011) and 50 in *Amblyomma sculptum* (Moreira et al., 2017). Two groups described 18 and 22 serpins in *R. microplus*, respectively (Tirloni et al., 2014b; Rodriguez-Valle et al., 2015) and at least 36 serpins were found in several published trancriptomes from *I. ricinus* (our own unpublished data based on the analysis of transcriptomes) (Schwarz et al., 2013; Kotsyfakis et al., 2015a,b; Perner et al., 2016). Another recent publication described 10 different serpin transcripts in the sialotranscriptome of the tick *Hyalomma excavatum* (Ribeiro et al., 2017). Despite high number of identified transcripts, only small portion was characterized functionally.

#### Tick serpins with known function

To date, almost 20 tick serpins from different tick species have been functionally validated by *in vitro* assays, *in vivo* experimental models, vaccination and by RNA interference (RNAi) experiments (Table 1). These are detailed below.

**Table 1:** **Tick serpins with known function**.

**Serpin**	**Tick species**	**Secreted**	**Effect (where known)**	**Tissue/stage**	**Target enzyme**	**References**
AamS6	*A. americanum*	Yes	Reduced platelet aggregation and delayed plasma clotting time	SG, MG, OVA, CA	Plasmin, papain, elastase, chymase	Chalaire et al., 2011; Mulenga et al., 2013
AAS19	*A. americanum*	Yes	Anti-coagulant protein, delayed clotting in recalcification and thrombin time assays		Trypsin, plasmin, fIXa, fXa, fXIa, fXIIa, thrombin, tryptase, chymotrypsin	Kim et al., 2015; Porter et al., 2015
			RNAi led to smaller blood meals and deformed ticks	SG, MG, OVA, SYN, CA, MT		Kim et al., 2016
			Feeding on immunized rabbits led to smaller blood meals and disrupted egg laying			
HLS1	*H. longicornis*	No	Feeding on immunized animals increased tick mortality rate	MG		Sugino et al., 2003
HLS2	*H. longicornis*	No	Prolonged coagulation, Immunization of rabbits increased tick mortality	LY		Imamura et al., 2005
Ipis-1	*I. persulcatus*	Yes	Inhibited proliferation and IFN-γ production of bovine PBMCs	SG		Toyomane et al., 2016
Iris	*I. ricinus*	Yes	Disrupted blood coagulation and fibrinolysis	SG, saliva	Elastase, thrombin, t-PA, fXa, trypsin	Prevot et al., 2006
			Suppressed T cell and splenocyte proliferation			Leboulle et al., 2002a
			Altered cytokine secretion by PBMC			Prevot et al., 2009
			Bound monocytes/macrophages and inhibited TNF secretion			Prevot et al., 2007
			Vaccination resulted in higher mortality and lower engorgement			
IRS-2	*I. ricinus*	Yes	Inflammation inhibitor, bound mast cell protease-4, blocked induced platelet aggregation	SG, OVA, MG	Cathepsin G, chymase, thrombin, trypsin, a-chymotrypsin	Chmelar et al., 2011
			Inhibited Th17 differentiation by reduced production of IL-6 in DC			Palenikova et al., 2015
IxscS-1E1	*I. scapularis*	Yes	Inhibited platelet aggregation and plasma clotting	SG, MG	Thrombin, trypsin, cathepsin G, fXa	Mulenga et al., 2009; Ibelli et al., 2014
RAS-1, 2	*R. appendiculatus*	No	Feeding on immunized animals increased tick mortality rate			Imamura et al., 2006
RAS-3, 4	*R. appendiculatus*	Yes	Feeding on immunized animals increased tick mortality rate and delayed *Theleria* infection			Imamura et al., 2008
RHS-1	*R. haemaphysaloides*	Yes	Anticoagulation activity, RNAi disrupted tick feeding	SG	Chymotrypsin, thrombin, fXa	Yu et al., 2013
RHS-2	*R. haemaphysaloides*	No	RNAi disrupted tick feeding	MG	Chymotrypsin, thrombin, fXa	Yu et al., 2013
RmS-3	*R. microplus*	Yes	Reduced platelet aggregation Feeding of ticks with RMS-3 antibodies impaired reproduction	SG, MG, CA	Chymotrypsin, cathepsin G, elastase, chymase	Tirloni et al., 2014b, 2016; Rodriguez-Valle et al., 2012, 2015
RmS-6	*R. microplus*	Yes		SG, MG, OVA, CA	Trypsin, plasmin, fXa, fXIa, chymotrypsin	Tirloni et al., 2014b, 2016
RmS-15	*R. microplus*	Yes	Delayed plasma clotting	SG, OVA, CA	Thrombin	Tirloni et al., 2014b; Xu et al., 2016
RmS-17	*R. microplus*	Yes	Delayed plasma clotting, reduced platelet aggregation	SG, MG, OVA, CA	Trypsin, plasmin, cythepsin G, chymotrypsin, fXIa	Tirloni et al., 2014b, 2016
rSerpin	*R. microplus*	Yes	Feeding on immunized animals increased feeding time and tick mortality and reduced tick engorgement and egg mass	SG		Kaewhom et al., 2007; Jittapalapong et al., 2010

*SG, salivary glands; MG, midgut; OVA, ovaries; FB, fat body; HE, hemocytes; MAL, Malpighian tubules; DC, dendritic cells*.

##### AamS6 (A. americanum)

Only two serpins (AamS6 and AAS19) have been characterized thus far in *A. americanum*, despite the overall high number of serpins identified in this tick (Porter et al., 2015). *A. americanum* serpin 6 (AamS6) is upregulated during first 3 days of feeding and is likely to be injected into the host during feeding; however, RNAi did not affect tick feeding ability (Chalaire et al., 2011). Recombinant AamS6 inhibited the serine proteases trypsin, chymotrypsin, elastase, and chymase and the cysteine protease papain in a dose-dependent manner (Chalaire et al., 2011). AamS6 also reduced platelet aggregation and delayed plasma clotting time, suggesting that this serpin facilitates blood feeding by ticks (Mulenga et al., 2013). The complement activation pathway, however, was not affected (Mulenga et al., 2013).

##### AAS19 (A. americanum)

AAS19 is an anti-coagulant that was shown to inhibit five of the eight serine protease blood clotting factors. AAS19 inhibited thrombin—but not ADP—and cathepsin G-activated platelet aggregation and delayed clotting in re-calcification and thrombin time assays (Kim et al., 2015). AAS19 RNAi halved the blood intake and resulted in morphological deformation of ticks (Kim et al., 2016). In rabbits, immunized with AAS19, tick feeding was faster, but smaller blood volumes were ingested, and tick ability to lay eggs was impaired (Kim et al., 2016).

##### HLS-1 and 2 (Haemaphysalis longicornis)

Sugino and colleagues isolated a serpin from *H. longicornis* in 2003 (HLS1) (Sugino et al., 2003). Recombinant HLS1 displayed anticoagulant activity, and nymph and adult tick feeding on immunized rabbits resulted in 43.9 and 11.2% tick mortality, respectively. Antibodies raised against tick saliva did not react with recombinant HSL1, suggesting that the serpin was not secreted (Sugino et al., 2003). Moreover, HLS1 expression was detected in the midgut rather than the salivary glands, and HLS1 was therefore considered a concealed antigen, similar to the first commercially used anti-tick vaccine based on the Bm86 tick protein (Willadsen et al., 1995). HLS1 does not contain a signal peptide. Therefore, it is likely that HLS1 is not a secreted protein playing an immunomodulatory or anti-hemostatic role in the host during tick feeding.

A second serpin from *H. longicornis* (HLS2) possesses a signal sequence and seems to be secreted by hemocytes into the haemolymph but not by the salivary glands or midgut (Imamura et al., 2005). HLS2 prolonged the coagulation time in a dose-dependent manner (Imamura et al., 2005), and rabbit vaccination with HLS2 resulted in greater immunization than with HLS1 and almost 50% mortality of feeding nymphs and adults (Imamura et al., 2005). This might be explained by better accessibility and inactivation of extracellular HLS2 in the haemolymph by antibodies from the ingested blood of immunized animals.

##### Ipis-1 (Ixodes persulcatus)

To date, Ipis-1 is the only characterized salivary serpin from tick *I. persulcatus* (Toyomane et al., 2016). Ipis-1 transcripts were detected only in salivary glands of ticks at same level throughout all phases of feeding. It significantly reduced IFN-γ production and the proliferation of bovine PBMC cells after ConA stimulation. Authors suggest that Ipis-1 could inhibit T cells function by direct interaction with this cell population (Toyomane et al., 2016).

##### Iris (I. ricinus)

The first tick serpin to be described that had an effect on host defense mechanisms was named Iris (*Ixodes ricinus* immunosuppressor) (Leboulle et al., 2002a,b). Iris displayed several notable and important features. First, Iris was noted to inhibit T cell and splenocyte proliferation and altered peripheral blood mononuclear cell (PBMC)-derived cytokine levels (Leboulle et al., 2002a). Second, Iris showed anti-hemostatic properties including suppression of coagulation and fibrinolysis (Prevot et al., 2006). Finally, Iris was shown to bind to monocytes/macrophages and suppress the secretion of TNF (Prevot et al., 2009). Interestingly, these activities were independent on the protease inhibitory function of Iris. Of note, Iris, together with HLS1 and several other proteins, belongs to a group of serpins in *Ixodes* spp. that have methionine and cysteine in their reactive center loop (RCL) and lack a signaling peptide, suggesting intracellular rather than extracellular function. However, Iris has been detected in tick saliva using a polyclonal serum raised against recombinant protein (Leboulle et al., 2002a; Prevot et al., 2007), and vaccination of rabbits with recombinant Iris increased the mortality of feeding ticks and lowered weight after engorgement (Prevot et al., 2007). This contradictory observation might be explained by cross-reactivity with another secreted serpin or by the action of another, non-classical secretory mechanism (Nickel, 2003). Nevertheless, Iris represents a pleiotropic protein that affects multiple processes simultaneously via independent mechanisms.

##### IRS-2 (I. ricinus)

IRS-2 (*Ixodes ricinus* serpin-2) was the second serpin to be characterized in *I. ricinus*. IRS-2 has tryptophan in its P1 site, confirmed by its resolved crystal structure (Kovarova et al., 2010; Chmelar et al., 2011). IRS-2 displayed inhibitory specificity against mast cell chymase and cathepsin G, two proteases involved in inflammatory responses (Chmelar et al., 2011), with its anti-inflammatory function evidenced by *in vivo* paw edema experiments, in which IRS-2 significantly decreased paw swelling and neutrophil recruitment in treated animals (Chmelar et al., 2011). Moreover, IRS-2 inhibited the production of proinflammatory cytokine IL-6 in dendritic cells (DC) and impaired IL-6-dependent JAK/STAT3 signaling in T-helper (Th) cells, inhibiting the maturation of proinflammatory Th17 lymphocytes (Palenikova et al., 2015). IRS-2 also inhibited platelet aggregation induced by cathepsin G but not other inducers such as collagen or arachidonic acid derivatives (Chmelar et al., 2011).

##### IxscS-1E1 (I. scapularis)

A blood meal-induced salivary serpin IxscS-1E1 from *I. scapularis* has been shown to trap thrombin and trypsin in SDS- and heat-stable complexes, reduce their activity and inhibit the activities of cathepsin G and factor Xa, although protease/inhibitor complexes were not detected (Ibelli et al., 2014). Furthermore, IxscS-1E1 inhibited adenosine diphosphate- and thrombin-activated platelet aggregation and delayed plasma clotting time, suggesting an anti-hemostatic role (Ibelli et al., 2014). IxscS-1E1 had no effect on the classical complement activation pathway (Ibelli et al., 2014).

##### RAS-1, 2, 3, 4 (Rhipicephalus appendiculatus)

Four serpin cDNAs, two putatively secreted (RAS-3 and RAS-4) and two putatively intracellular (RAS-1 and RAS-2), were identified in and isolated from the salivary glands of *R. appendiculatus* (Mulenga et al., 2003). Although RAS-1 and RAS-2 are expressed in the salivary glands, antibodies against them were not found at the bite site as determined by the reactivity of anti-tick saliva sera to recombinant RAS-1 and RAS-2 (Imamura et al., 2006). This finding is, however, consistent with their predicted intracellular location (Imamura et al., 2006). Vaccination of cattle with a RAS-1/RAS-2 cocktail resulted in a 61.4% reduction in nymph engorgement rate and a 28 and 43% increase in mortality rate in female and male adult ticks, respectively (Imamura et al., 2006). Similar results were obtained when cattle were vaccinated with a mixture of two secreted serpins RAS-3 and RAS-4 and a 36-kDa immunodominant cement protein RIM36 (Imamura et al., 2008): immunization resulted in 40% mortality rate for *R. appendiculatus* ticks and almost 50% for *Theileria parva*-infected female ticks (Imamura et al., 2008). However, no significant protective effect against infection with *T. parva* was observed in spite of a 1–2 day delay in the detection of pathogens in the host peripheral blood after immunization (Imamura et al., 2008).

##### RHS-1 and 2 (Rhipicephalus haemaphysaloides)

Two serpins (RHS-1 and RHS-2) have been identified and characterized from *R. haemaphysaloides* (Yu et al., 2013), both of which were expressed in the salivary glands and midguts of ticks fed for 4 days. Both inhibited chymotrypsin, and RHS-1 also inhibited thrombin (Yu et al., 2013). Consistent with their inhibitory activity, only RHS-1 exhibited anticoagulation activity based on the activated partial thrombin time assay (Yu et al., 2013). Only RHS-1 seems to be secreted into the saliva and the host, as only RHS-1 was detected by serum from rabbits that were exposed to ticks and only RHS-1 possesses a signal peptide sequence (Yu et al., 2013). Nevertheless, RNAi of both serpins negatively affected the attachment rate after 24 h and decreased the engorgement rate (Yu et al., 2013).

##### RmS-3, 6, 15, 17 (R. microplus)

Serpin RmS-3 from *R. microplus* displayed anti-elastase and anti-chymotrypsin inhibitory activities (Rodriguez-Valle et al., 2015). Tirloni and colleagues subsequently confirmed this specificity (albeit with much lower inhibitory activity), tested more proteases, and found the highest inhibitory activity against chymase and cathepsin G (Tirloni et al., 2016). RmS-3 is likely to be secreted into the saliva and the host as evidenced by differential antibody responses of tick-resistant and tick-susceptible cattle (Rodriguez-Valle et al., 2012). RmS-3 is expressed in nymphs and in the salivary glands of adult ticks, data on RmS-3 transcription in ovaries differ between the two studies (Tirloni et al., 2014b; Rodriguez-Valle et al., 2015). Capillary feeding of ticks with a RmS-3 antibody reduced tick reproductive capacity (Rodriguez-Valle et al., 2012, 2015).

In addition to RmS-3, three other recombinant *R. microplus* serpins were produced for enzymatic and functional characterization (Tirloni et al., 2014a,b; Xu et al., 2016). RmS-6 inhibited factor Xa, factor XIa and plasmin, suggesting an anticoagulant function, while RmS-17 showed weaker inhibitory activity against chymotrypsin, cathepsin G, trypsin, and plasmin (Tirloni et al., 2016). Both RmS-3 and RmS-17 inhibited cathepsin G-induced platelet aggregation. Interestingly, RmS-3, -6, and -17 from *R. microplus* were recognized by antibodies raised by the saliva of *A. americanum, I. scapularis*, and *Rhipicephalus sanguineus*, suggesting a potential use for these proteins as an universal tick vaccine (Tirloni et al., 2016) but also highlighting the pitfall of false-positive detection of serpins in tick saliva. RmS-15 was identified as a thrombin inhibitor and, together with RmS-17, delayed plasma clotting in a re-calcification time assay (Tirloni et al., 2016; Xu et al., 2016). Moreover, RmS-15 is an immunogen, as the infestation of cattle with *R. microplus* resulted in increased anti-RmS-15 IgG titers (Xu et al., 2016).

##### rSerpin (R. microplus)

Rabbits immunized with putatively secreted serpin (rSerpin) from *R. microplus* (Kaewhom et al., 2007) led to extended feeding time, an 83% reduction in adult engorgement, 67% mortality of engorged females and a 34% reduction in egg mass weight (Jittapalapong et al., 2010).

### Cystatins

Cystatins form a superfamily of tight-binding reversible inhibitors of papain-like cysteine proteases and legumains and, similar to serpins, they are present in all organisms including prokaryotes (Kordis and Turk, 2009). Cystatins regulate many physiological processes including immunity-related mechanisms such as antigen presentation, phagocytosis, and cytokine expression (Zavasnik-Bergant, 2008). There are four cystatin subgroups: type 1 (stefins), type 2, type 3 (kininogens), and type 4 cystatins (fetuins) (Rawlings and Barrett, 1990). Cystatins' target proteases are usually lysosomal cathepsins involved in protein degradation, but they also target those involved in degradation of antigens presented via MHCII to lymphocytes or in the activation of caspase 1 and thus inflammasome regulation (Jin and Flavell, 2010; Turk et al., 2012).

#### Cystatins with known function

Similarly to serpins, there are around 20 tick cystatins described in the literature and only type 1 and type 2 cystatins have thus far been reported in ticks. While stefins lack a secretory signal and are most likely involved in the intracellular digestion of hemoglobin or in developmental processes, type 2 cystatins are secreted and expressed in both the midgut and salivary glands (Schwarz et al., 2012). Tick cystatins either regulate hemoglobin digestion, which is driven by cathepsins (Horn et al., 2009), or they can be secreted as immunomodulators into the host with saliva. The majority (84%) of tick cystatin transcripts that are conserved across tick species, belong to the extracellular group, suggesting predominantly immunomodulatory role (Ibelli et al., 2013) Tick cystatins with experimentally validated functions are listed in Table 2 and detailed below.

**Table 2:** **Tick cystatins with known function**.

**Cystatin**	**Tick species**	**Secreted**	**Effect (where known)**	**Tissue/stage**	**Target enzyme**	**References**
Bmcystatin	*R. microplus*	No		SG, OVA, FB	Cathepsin L, VDTCE	Lima et al., 2006
BrBmcys2a	*R. microplus*	Yes		MG, OVA, FB		Imamura et al., 2013
BrBmcys2b	*R. microplus*	Yes		MG	Cathepsin B, C, L	Imamura et al., 2013; Parizi et al., 2015
BrBmcys2c	*R. microplus*	Yes		MG	Cathepsin C, L	Imamura et al., 2013; Parizi et al., 2015
BrBmcys2d, e	*R. microplus*	Yes		larvae		Imamura et al., 2013
Cystatin	*A. americanum*	Yes	RNAi caused decreased tick body weight, dying of ticks during feeding or disrupted feeding to the fully engorged state	MG, SG		Karim et al., 2005
HISC-1	*H. longicornis*	Yes		SG	Cathepsin L, papain	Yamaji et al., 2009b
Hlcyst-1	*H. longicornis*	No	Regulated hemoglobin degradation	MG	Cathepsin B, H, L, papain, HlCPL-A	Zhou et al., 2006b, 2009; Yamaji et al., 2009a, 2010
Hlcyst-2	*H. longicornis*	Yes	Regulated hemoglobin degradation, inhibited *Babesia* growth *in vitro*	MG, SG, OVA, HE, FB	Cathepsin L, papain, HlCPL-A	Zhou et al., 2006b; Yamaji et al., 2009a, 2010
Hlcyst-3	*H. longicornis*	Yes		MG, SG, OVA, HE, FB	Cathepsin L, papain	Zhou et al., 2006b, 2010
JpIocys2	*I. ovatum*	Yes		Assumed MG	Cathepsin B, C, L	Parizi et al., 2015
JpIpcys2a, b, c	*I. persulcatus*	Yes		SG, MG / larvae, nymphs, adult	Cathapsin L, papain	Rangel et al., 2017
Om cystatin 1	*O. moubata*	Yes		MG	Cathepsin B, C, H	Grunclova et al., 2006
Om cystatin 2	*O. moubata*	Yes	Inhibited TNF-α and IL-12 production by DC and proliferation of CD4+ T cells, immunization decreased tick feeding success	SG, OVA, MAL, MG	Cathepsin B, C, H, L, S, papain	Grunclova et al., 2006; Kotsyfakis et al., 2010
RHcyst-1	*R. haemaphysaloides*	No	Inhibitors, RNAi of RHcyst-1 impaired tick attachment rate and decreased hatching rate	Egg, larvae	Cathepsin B, C, H, L, S, papain	Wang et al., 2015b
RHcyst-2	*R. haemaphysaloides*	Yes		Egg, adult MG, SG, OVA, FB	Cathepsin B, C, H, L, S, papain	Wang et al., 2015a
Rmcystatin3	*R. microplus*	Yes		FB, HE	Cathepsin B, L, BmCl1	Lu et al., 2014
Sialostatin L	*I. scapularis*	Yes	Inhibited CTL proliferation, anti-inflammatory effects	SG	Cathepsin C, L, V, X, papain	Valenzuela et al., 2002
			Impaired DC maturation and differentiation and T cells proliferation		Binds cathepsin S	Kotsyfakis et al., 2006
			Prevented experimental asthma, inhibited IL-9 production by Th9 cells and mast cells by targeting IRF-4			Sa-Nunes et al., 2009
			Decreased IFN-β production in DC and DC maturation			Horka et al., 2012; Klein et al., 2015
			Attenuated IFN-β-triggered JAK/STAT signaling pathway in dendritic cells			Lieskovska et al., 2015a
						Lieskovska et al., 2015b
Sialostatin L2	*I. scapularis*	Yes	RNAi caused tick mortality, reduced weight and less eggs	SG, MG	Cathepsin C, L, S, V	Kotsyfakis et al., 2007
			Immunization caused decreased feeding ability of nymphs			Kotsyfakis et al., 2008
			Enhanced establishment of *Borrelia* infection			Kotsyfakis et al., 2010
			Inhibited caspase-1 maturation and diminished IL-1β and IL-18 secretion by macrophages during *Anaplasma phagocytophilum* infection			Chen et al., 2014
			Attenuated IFN-β-triggered JAK/STAT signaling in DC and promotes TBEV replication, decreases MIP-a and IP-10 production by DC			Lieskovska et al., 2015a,b

*SG, salivary glands; MG, midgut; OVA, ovaries; FB, fat body; HE, hemocytes; MAL, Malpighian tubules; VDTCE, vitellin-degrading cysteine endopeptidases; DC, dendritic cell; TBEV, tick-borne encephalitis virus*.

##### Bmcystatin (R. microplus)

Bmcystatin from *R. microplus* is specifically expressed in the salivary glands, ovaries, and fat bodies. Bmcystatin did not inhibit papain but inhibited human cathepsin L and tick vitellin-degrading cysteine endopeptidase (VDTCE), suggesting a role in regulating tick embryogenesis (Lima et al., 2006).

##### BrBmcys2a, b, c, d, e, (R. microplus)

In addition to Bmcystatin, another five cystatins (BrBmcys2a, b, c, d, e) were identified in the cattle tick *R. microplus*. Their expression differs among various developmental stages and tissues, but since their presence has only been assessed by immunodetection methods, cross reactivity between antibodies is possible and has indeed been reported (Imamura et al., 2013). This study also examined the inhibitory specificity of two cystatins: while BrBmcys2b targeted cathepsins B, C, and L, BrBmcys2c only inhibited cathepsins C and L (Parizi et al., 2015). Antibodies raised against recombinant proteins detected BrBmcys2b in all tick tissues, while anti-BrBmcys2c serum only recognized the protein in the gut from partially engorged females and in the ovaries, salivary glands, and fat bodies from fully engorged females (Parizi et al., 2015). The expression patterns suggest rather homeostatic function of these cystatins in ticks than immunomodulatory activity in the host (Imamura et al., 2013).

##### Cystatin (A. americanum)

One cystatin was detected in the salivary glands and midguts of unfed and partially fed *A. americanum* ticks (Karim et al., 2005). RNAi of this cystatin led to a 90 and 50% reduction in transcript abundance in the early and late phases of feeding, respectively. RNAi knockdown decreased tick body weight, killed ticks during feeding, and disrupted feeding to full engorgement. Rabbits pre-exposed to dsRNA-injected ticks were re-exposed to naïve ticks, which led to detachment of 34% ticks after 1 day and over 50% mortality of attached ticks (Karim et al., 2005). No such effect was observed in the control group, in which rabbits were pre-exposed to normal ticks. Such a strong immune response indicates an important immunomodulatory function for silenced cystatin that impairs responses to salivary antigens and leads to an overall less intense immune reaction (Karim et al., 2005).

##### HISC-1 (H. longicornis)

HISC-1 is a type 2 cystatin detected in *H. longicornis* (Yamaji et al., 2009b). It is found mainly in the acinar cells of the tick salivary glands and is therefore likely to be secreted into the host. The number of transcripts was found to be approximately 5-fold higher in the salivary glands than in the midgut, with strong upregulation in early phase of blood feeding and with a pattern suggestive of importance in the feeding process. HISC-1 inhibited cathepsins L and papain but not cathepsin B (Yamaji et al., 2009b).

##### Hlcyst-1, 2 and 3 (H. longicornis)

While Hlcyst-1 is a type 1 intracellular cystatin with specificity against papain and cathepsin L (Zhou et al., 2009), Hlcyst-2 and Hlcyst-3 are secreted type 2 cystatins (Zhou et al., 2006b, 2010). Hlcyst-2 has been shown to inhibit cathepsin L and cathepsin B, with transcripts found mainly in the midgut and hemocytes of all tick developmental stages. Expression increased with tick development and was induced by blood feeding (Zhou et al., 2006b). Moreover, Hlcyst-2 expression was induced by injecting ticks with LPS or *Babesia gibsoni*, suggesting a role in tick immunity. *In vitro* cultivation of *B. gibsoni* in the presence of Hlcyst-2 significantly inhibited pathogen growth (Zhou et al., 2006b). Hlcyst-1 and Hlcyst-2 also inhibited cysteine protease HlCPL-A with hemoglobinase activity, isolated from *H. longicornis*, which can act as natural target of these cystatins, suggesting an involvement of both the protease and its inhibitors in blood digestion (Yamaji et al., 2009a). Hlcyst-3 inhibited papain and cathepsin L, and its expression was detected preferentially in the midgut (Zhou et al., 2010).

##### JpIocys2 (Ixodes ovatum)

JpIocys2 was isolated from *I. ovatum* and was shown to modulate the enzymatic activity of cathepsins B, C, and L with cathepsin L as the preferred target (Parizi et al., 2015). Similar to BrBmcys2b and BrBmcys2c, JpIocys2 is considered to be involved in tick homeostasis and egg development.

##### JpIpcys2a, b, c (I. persulcatus)

Three novel cystatins from *I. persulcatus*, JpIpcys2a, b, and c, have recently been described in terms of sequence and structural analysis and expression profile (Rangel et al., 2017). All three possess a signal peptide and two disulfide bridges in their mature form. Although varying in their tertiary structure, all three *I. persulcatus* cystatins should bind human cathepsin L and papain, based on *in silico* analyses. Transcripts of all three cystatins were detected in almost all tissues (salivary glands, midgut, carcass) and stages (larvae, nymphs, adults) of tick development. The only exception was absence of JpIpcys2c transcripts in unfed larvae. Furthermore, vaccination of hamsters with a structurally similar BrBmcys2c cystatin from *R. microplus* did not show any cross-reactivity and did not lead to impaired *I. persulcatus* feeding or reproduction (Rangel et al., 2017).

##### Om-cystatin 1 and 2 (Ornithodoros moubata)

Om-cystatin 1 and 2 were described in a soft tick *O. moubata* (Grunclova et al., 2006). While Om-cystatin 1 transcripts were found only in the midguts of unfed ticks, Om-cystatin 2 mRNA was present in all tissues. Transcript levels were rapidly suppressed after tick feeding. Both possessed inhibitory activity against cathepsins B, C, and H and papain (Grunclova et al., 2006). Om-cystatin 2 was further functionally and structurally characterized under the name OmC2 (Salát et al., 2010). OmC2 inhibited the secretion of pro-inflammatory cytokines TNF and IL-12 by DC after LPS stimulation and reduced antigen-specific CD4^+^ T cell proliferation induced by DC (Salát et al., 2010). Exposing OmC2 immunized mice to *O. moubata* nymphs reduced feeding ability and increased mortality during nymphal development to the next stage. Interestingly, nymphs mortality was positively correlated with higher titers of anti-OmC2 antibodies in the serum (Salát et al., 2010).

##### RHcyst-1 and RHcyst-2 (R. haemaphysaloides)

Two cystatins have been described in *R. haemaphysaloides*, RHcyst-1 and RHcyst-2. RHcyst-1 is an intracellular type 1 cystatin that inhibited cathepsins L, B, C, H, and S and papain, with strongest affinity to cathepsin S (Wang et al., 2015b). RHcyst-1 was expressed at all developmental stages but was most abundant in tick eggs, and its expression decreased throughout the development. RNAi of RHcyst-1 reduced the attachment rate of adult ticks and decreased hatching rate (Wang et al., 2015b). RHcyst-2 is a secreted type 2 cystatin that inhibited the same cathepsins as RHcyst-1 (Wang et al., 2015a) and was again present at all developmental stages with highest expression in eggs. However, RHcyst-2 expression increased during blood feeding, and RHcyst-2 was secreted to the host during tick feeding according to immunodetection methods (Wang et al., 2015a).

##### Rmcystatin3 (R. microplus)

Rmcystatin3 inhibited cathepsins L and B and *Boophilus microplus* cathepsin L-1 (BmCl1) (Lu et al., 2014). Bmcystatin3 transcripts were found in tick hemocytes, fat bodies, and salivary glands, but protein was only detected in hemocytes and the fat bodies by western blotting. Infection of ticks with *E. coli* significantly downregulated Bmcystatin3 expression (Lu et al., 2014) but increased efficacy of pathogen clearance, suggesting that Rmcystatin3 may be a negative regulator of tick immune responses, probably by regulating cysteine proteases responsible for the production of antimicrobial effectors in hemocytes (Lu et al., 2014).

##### Sialostatin L (I. scapularis)

One of the best studied tick cystatins is sialostatin L, a type 2 cystatin detected in *I. scapularis*. Sialostatin L has preferential specificity for cathepsin L; however, cathepsins V, C, X, S, and papain were also inhibited in enzymatic assays (Kotsyfakis et al., 2006). In the same study, sialostatin L inhibited the proliferation of the cytotoxic T lymphocyte cell line CTLL-2, suggesting its effect on adaptive immunity. Moreover, the anti-inflammatory activity of sialostatin L was confirmed in a mouse model of carrageenan-induced paw edema, in which sialostatin L reduced edema and neutrophil myeloperoxidase activity (Kotsyfakis et al., 2006).

Sialostatin L has been shown to inhibit IL-2 and IL-9 production by Th9 lymphocytes (Horka et al., 2012). IL-9 production by Th cells is IL-2 dependent (Schmitt et al., 1994), but the addition of exogenous IL-2 did not rescue IL-9 synthesis, suggesting that mechanisms other than IL-2 reduction may be involved in IL-9 inhibition (Horka et al., 2012). Nevertheless, the impairment of Th9 cells by sialostatin L abrogated the eosinophilia and airway hyperresponsiveness of mice challenged with OVA antigen (Horka et al., 2012). The inhibition of IL-9 production together with reduced expression of IL-1β and IRF4 (interferon regulating factor 4) was also observed in mast cells, with IL-9 production rescued by the application of exogenous IL-1β (Klein et al., 2015). The inhibition of IL-9 was IRF4 or IL-1β dependent, as proven by the fact that IRF4-deficient or IL-1 receptor-deficient mast cells failed to produce IL-9. The transcription factor IRF4 binds to IL-1β and IL-9 promoters, implying that sialostatin L inhibits IL-9 production via its effect on IRF4 (Klein et al., 2015). Furthermore, mice with IRF4 knockdown in mast cells or mice administered with sialostatin L showed a strong reduction in eosinophilia and airway hyperresponsiveness, important symptoms of asthma. Conversely, sialostatin L did not affect mast cell degranulation or IL-6 expression (Klein et al., 2015).

Sialostatin L inhibits cathepsin S, resulting in reduced antigen-specific CD4^+^ T cell proliferation *in vitro* and *in vivo*; sialostatin L treatment during OVA immunization impaired early T cell expansion of splenocytes in OT-II mice and late recall immune responses by impairing the proliferation of lymph node cells (Sa-Nunes et al., 2009). Sialostatin L also potently prevented symptoms of experimental autoimmune encephalomyelitis in mice accompanied by impaired IFN-γ and IL-17 production and specific T cell proliferation (Sa-Nunes et al., 2009).

In addition to modulating T cells, sialostatin L inhibited DC maturation and reduced the production of IL-12 and TNF by DC (Sa-Nunes et al., 2009). These effects on DC can also be attributed to anti-cathepsin S activity, as cathepsin S plays a role in an invariant chain processing (Pierre and Mellman, 1998) and its inhibition thus leads to poor antigen presentation by DC (Sa-Nunes et al., 2009). Similar to another *I. scapularis* cystatin Sialostatin L2 (Lieskovska et al., 2015b), sialostatin L attenuated IFN-β-triggered JAK/STAT signaling in DC (Lieskovska et al., 2015a). However, unlike Sialostatin L2, it did not suppress expression of the IP-10 chemokine or IRF-7, suggesting that these two cystatins can produce the same phenotype by impairing different pathways in the same cell (Chmelar et al., 2016). It also decreased IFN-β production in DC activated by either *Borrelia* or TLR-7 ligand (Lieskovska et al., 2015a).

##### Sialostatin L2 (I. scapularis)

Sialostatin L2 is an *I. scapularis* cystatin similar in sequence to sialostatin L but with different anti-protease potency, antigenicity, and expression pattern. Unlike sialostatin L, sialostatin L2 transcripts accumulate in the salivary glands during tick feeding (Kotsyfakis et al., 2007). Its target proteases are cathepsins L, V, S, and C with preferential affinity for cathepsins L and V (Kotsyfakis et al., 2007). Sialostatin L2 was shown to inhibit inflammasome formation during infection with *A. phagocytophilum* (Chen et al., 2014) via sialostatin L2-driven inhibition of caspase-1 maturation, leading to diminished IL-1β and IL-18 secretion by macrophages after stimulation with *A. phagocytophilum* (Chen et al., 2014). However, the mechanism was not due to direct caspase-1 or cathepsin L inhibition, but was instead dependent on reactive oxygen species (ROS) production by NADPH oxidase that was affected by the Loop2 domain of the cystatin (Chen et al., 2014). As mentioned above, sialostatin L2 interfered with JAK/STAT signaling in DC (Lieskovska et al., 2015b), attenuating STAT phosphorylation upon IFN-β treatment and thus inhibiting the IFN-β stimulated IP-10 and IRF7 chemokine genes (Lieskovska et al., 2015b). No interference with the IFN-β receptor was observed, so the downstream components of the pathway were most likely affected. Moreover, this activity enhanced the replication of tick borne encephalitis virus in DC (Lieskovska et al., 2015b). Sialostatin L2 decreased the production of specific DC chemokines MIP-1α and IP-10 in response to *Borrelia* (Lieskovska et al., 2015a). Upon LTA/TLR2 stimulation of DC, sialostatin L2 attenuated Erk1/2 phosphorylation, inhibited the PI3K pathway by reducing Akt phosphorylation, and also reduced NF-κB phosphorylation. Impaired Erk1/2 phosphorylation was the only effect observed for sialostatin L2 after stimulation of DC with *Borrelia* spirochetes (Lieskovska et al., 2015a).

The role of sialostatin L2 in *Borrelia* transmission and tick feeding has also been addressed. RNAi of sialostatin L2 led to 40% mortality in tick feeding, reduced tick size, and reduced the number of eggs by about 70% (Kotsyfakis et al., 2007). Similar effects were seen when *I. scapularis* nymphs were exposed to guinea pigs immunized with sialostatin L2 (Kotsyfakis et al., 2008). The rejection rate of nymphs fed on immunized animals was three times higher compared to controls, and the time needed to finish a blood meal was prolonged by approximately 1 day (Kotsyfakis et al., 2008). Moreover, IgG isolated from immunized animals reduced sialostatin L2 inhibitory activity against cathepsin L (Kotsyfakis et al., 2008). Of note, sialostatin L2 has been referred to as a “silent antigen,” meaning that corresponding antibodies cannot be found in naïve animals exposed to ticks despite an increased titer of specific antibodies in animals pre-immunized with recombinant protein. This can be explained by the amount of sialostatin L2 injected via the saliva into the host being too small to elicit a response (Kotsyfakis et al., 2008). Sialostatin L2 has also been shown to play an important role in *Borrelia* infection (Kotsyfakis et al., 2010). The skin of mice simultaneously injected with *Borrelia* and sialostatin L2 contained six-times more spirochetes than controls. Sialostatin L2 does not appear to bind spirochetes directly and had no effect on *Borrelia* growth *in vitro*, so the mechanism of *Borrelia* growth boost in skin remains unknown (Kotsyfakis et al., 2010).

## Protease inhibitors at the tick-host interface

**Figure 1:**
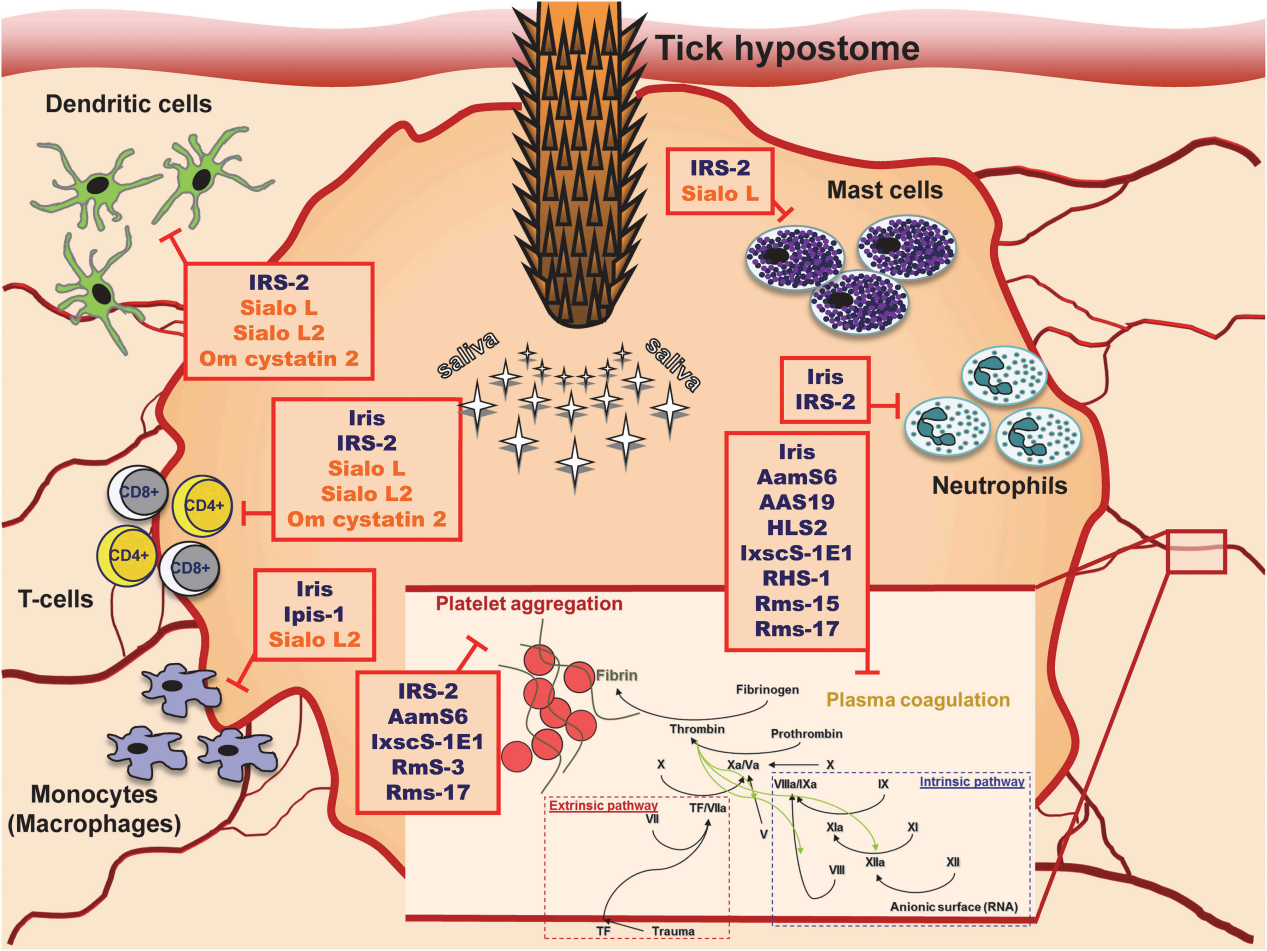
**Tick serpins and cystatins and their targets at the site of tick attachment**.

Tick cystatins and serpins can obviously affect many intracellular pathways and thus impair the functions of host immune cells. Moreover, they can also interfere with extracellular proteolysis, thereby inhibiting hemostasis (Figure 1). These activities take place at the site of attachment, where they cause local immunosuppression and inhibition of blood clotting. Of note, different inhibitors can cause similar phenotypes by targeting different pathways or even different components of the same pathway. Their actions are therefore redundant. Conversely, more than one effect is usually observed for a single inhibitor. Such concept of redundancy and pluripotency is probably a strategy developed by ticks during long-term co-evolution with their hosts (Chmelar et al., 2016). There is no doubt that salivary secretion at the tick-host interface is beneficial for the tick and deleterious for the host. From this perspective, tick inhibitors represent an important and interesting research field for the development of anti-tick vaccines and tick control strategies.

As shown on vaccination experiments, tick serpins and cystatins can contribute to the establishment of pathogens in the host (Imamura et al., 2008; Kotsyfakis et al., 2010). Such role of serpins is in accordance with observed positive effect of activated plasminogen activation system (PAS) with upregulated serpin PAI-2 on the establishment of *Borrelia burgdorferi* infection. The facilitation of infection resulted from direct enhancement of *Borrelia* dissemination and from the inhibition of inflammatory infiltration to the site of exposure (Haile et al., 2006). *Borrelia recurrentis* was shown to bind host serpin—C1 inhibitor—on its surface and thus inhibit complement activation (Grosskinsky et al., 2010). On contrary, mammalian cystatins were shown as regulators of cysteine proteases like cathepsin S and L, which contribute to the establishment of several viral infections (Kopitar-Jerala, 2012). Thus, the involvement of cystatins in the establishment of microbial and viral infection is not clear and cannot be easily addressed without experimental evidence.

## Tick protease inhibitors as novel drugs

### Cystatins

**Figure 2:**
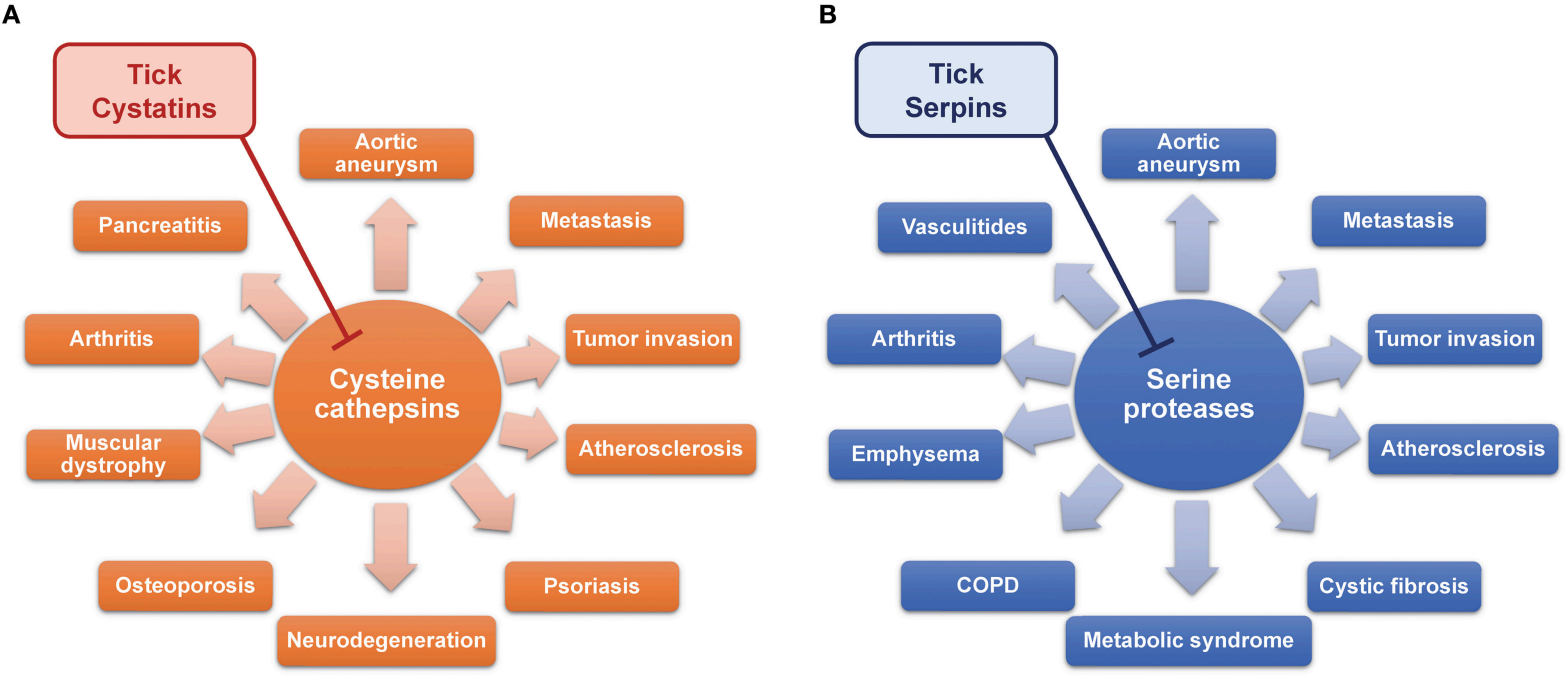
**The therapeutic potential of tick cystatins (A)** and serpins **(B)** by inhibiting cysteine and serine proteases in various diseases.

The inhibition of target proteases with tick-derived inhibitors can, however, be beneficial in different scenarios. Almost all the mammalian serine and cysteine proteases that are targets of tick inhibitors described in this review play important roles in various human diseases and pathologies. For a long time, the functions of lysosomal cysteine cathepsins (B, C, F, H, K, L, O, S, V, X, and W) were thought to be strictly limited to intracellular protein degradation and cellular metabolism. Recently, many cathepsins have been shown to be involved in multiple pathological processes. For example, increased serum levels of cathepsin L are associated with metastatic stage of different cancer types and poor patient prognosis (Tumminello et al., 1996; Chen et al., 2011). Tumor cells can produce high amounts of cathepsin L, leading to high serum level, which is considered as blood marker of cancer (Denhardt et al., 1987). High concentration of cathepsin L in tumor and its vicinity leads to extracellular matrix degradation, higher tumor invasiveness, and several cancer-related health complications (Sudhan and Siemann, 2015). Other cysteine cathepsins may also participate in tumor invasion and metastasis (Kuester et al., 2008; Tan et al., 2013), so cystatins are considered possible effectors that could block the deleterious activity of cysteine cathepsins in cancer (Cox, 2009; Hap et al., 2011). Cysteine cathepsins also contribute to neurodegenerative disorders such as Alzheimer's, Parkinson's, and Huntington's disease and amyotrophic lateral sclerosis (Figure 2A; Pislar and Kos, 2014). The leakage of lysosomal cathepsins induces neuronal apoptosis and can also increase the inflammatory milieu in the central nervous system (Pislar and Kos, 2014). Cysteine cathepsins are also implicated in the pathogenesis of psoriasis (Kawada et al., 1997), muscular dystrophy (Takeda et al., 1992), abdominal aortic aneurysm and atherosclerosis (Liu et al., 2006), osteoporosis and rheumatoid arthritis (Yasuda et al., 2005), and acute pancreatitis (Halangk et al., 2000). Relatively recent data are accumulating to suggest that cysteine cathepsins are promising therapeutic targets (Kos et al., 2014; Sudhan and Siemann, 2015). The wide spectrum of tick cystatins with varying specificities provides an opportunity to take advantage of this rich source of natural cathepsin inhibitors.

### Serpins

Serine proteases are best known as the building blocks of proteolytic cascades in the blood such as coagulation (Figure 1) or complement activation. The portfolio of their activities, however, is much wider. Neutrophils, mast cells, natural killer cells, and cytotoxic T cells all produce serine proteases responsible for extracellular matrix remodeling, microbe killing, cytokine activation, signaling via protease-activated receptors (PARs), or chemoattraction of leukocytes. As regulators of many processes, serine proteases often contribute to disease pathologies. Some diseases, in which serine proteases are implicated, are shown in Figure 2B. Signaling via PARs and the activation of coagulation in the tumor microenvironment link coagulation proteases with some of the complications seen in cancer (Shi et al., 2004; Han et al., 2011; Lima and Monteiro, 2013). Neutrophil proteases from azurophilic granules, namely cathepsin G, elastase, and protease 3 (PR3), play crucial roles in neutrophil anti-microbial activity and are indispensable for the clearance of some pathogens (Hahn et al., 2011; Steinwede et al., 2012). Many studies have also described neutrophil proteases as important regulators of inflammatory and immune processes (Pham, 2006, 2008), albeit with deleterious effects in some cases. For instance, due to the large amounts of elastin present in the lung connective tissue, lungs are very sensitive to dysregulation and/or increased levels of elastolytic proteases such as neutrophil elastase (Sandhaus and Turino, 2013), which results in several lung diseases. Elastase and cathepsin G facilitate the spreading of metastases to the lungs due to the degradation of antitumorigenic factor thrombospondin-1 (El Rayes et al., 2015). Furthermore, neutrophil proteases have been implicated in the pathogenesis of cystic fibrosis (Twigg et al., 2015; Wagner et al., 2016), chronic obstructive pulmonary disease (COPD) (Shapiro, 2002; Owen, 2008), and emphysema (Ekeowa et al., 2009). In anti-neutrophil cytoplasmic autoantibody (ANCA)-associated vasculitides such as Wegener's granulomatosis, neutrophils are activated by auto-antibodies against PR3 (Niles et al., 1989), leading to the production of neutrophil extracellular traps (NETs) containing PR3 and to necrosis (Kessenbrock et al., 2009). Cathepsin G is chemotactic for monocytes in rheumatoid arthritis (Miyata et al., 2007), and the inhibition of neutrophil elastase improved some of the symptoms of this disease (Di Cesare Mannelli et al., 2016). Interestingly, obesity and metabolic syndrome also seem to be affected by neutrophil proteases (Talukdar et al., 2012; Mansuy-Aubert et al., 2013). Mast cells are another significant source of several serine proteases, mainly chymases and tryptases, which are involved in extracellular matrix remodeling, chemoattraction of neutrophils, and protein processing and activation (Pejler et al., 2010). Mast cell chymase and tryptase have been shown to be involved in the pathogenesis of abdominal aortic aneurysm (Sun et al., 2009; Zhang et al., 2011) and atherosclerosis (Sun et al., 2007; Bot et al., 2015).

Due to these diverse and clinically relevant effects of serine proteases, their potential use as therapeutic targets is being thoroughly discussed by scientific community (Guay et al., 2006; Quinn et al., 2010; Caughey, 2016). Tick salivary glands express a large number of serine protease inhibitors with different specificities that could be used as novel drugs against malfunctioning proteases.

## Concluding remarks

Novel pharmacoactive compounds are being developed either by artificial synthesis or by isolating potential candidates from various organisms including parasites (Cherniack, 2011). For instance, hirudin (a thrombin inhibitor from leeches) and its congener bivalrudin have been useful in the treatment of blood coagulation disorders (Kennedy et al., 2012). Ticks are parasites that have evolved multiple ways to evade or manipulate host immune and hemostatic systems (Chmelar et al., 2012). Tick saliva contains hundreds of proteins not only with anti-hemostatic features (Maritz-Olivier et al., 2007) but also with anti-complement, anti-inflammatory, and immunomodulatory effects on the host (Kazimirova and Stibraniova, 2013).

As discussed in this review, salivary cystatins and serpins display such features and their functions have been studied thoroughly. Moreover, both superfamilies are represented in the vertebrate host and the functions of their members are often known. Therefore, we can predict at least to some degree, which processes or pathways will be targeted by tick proteins. An important advantage of cystatins and serpins is their functional specificity; for example, sialostatins L and L2 cause similar phenotypes (inhibition of IFN-β signaling) either by inhibiting the IFN-β production (sialostatin L) or by inhibiting STAT3 phosphorylation downstream from IFN-β (sialostatin L2) (Lieskovska et al., 2015a,b). The possibility of targeting specific processes is crucial for the development of “patient-tailored” immunotherapeutic strategies (Scherer et al., 2010; Stephenson et al., 2016). Furthermore, tick cystatins and serpins are not the only families in ticks that deserve attention, since there are many tick-specific proteins secreted into the saliva of unknown function. Characterizing ticks using the transcriptomic approach has created a broad field and data repository, which we can search for novel drugs and potential therapeutics.

## Author contributions

JC and JK wrote the manuscript, JK prepared the tables, JC prepared the figures, HL and MK edited and revised the manuscript.

### Conflict of interest statement

The authors declare that the research was conducted in the absence of any commercial or financial relationships that could be construed as a potential conflict of interest.
